# Distribution and identification of sand flies naturally infected with *Leishmania* from the Southeastern Peruvian Amazon

**DOI:** 10.1371/journal.pntd.0006029

**Published:** 2017-11-06

**Authors:** Victor Zorrilla, Maxy B. De Los Santos, Liz Espada, Rocío del Pilar Santos, Roberto Fernandez, Albino Urquia, Craig A. Stoops, Sarah-Blythe Ballard, Andres G. Lescano, Gissella M. Vásquez, Hugo O. Valdivia

**Affiliations:** 1 Department of Entomology, U.S. Naval Medical Research Unit 6, Lima, Peru; 2 Department of Parasitology, U.S. Naval Medical Research Unit 6, Lima, Peru; 3 Dirección Regional de Salud Madre De Dios, Ministerio de Salud, Puerto Maldonado, Peru; 4 Facultad de Salud Pública, Universidad Peruana Cayetano Heredia, Lima, Peru; University of Notre Dame, UNITED STATES

## Abstract

**Background:**

Cutaneous leishmaniasis (CL) is an important health problem in the New World affecting civilian and military populations that are frequently exposed in endemic settings. The Peruvian region of Madre de Dios located near the border with Brazil is one of the most endemic CL regions in South America with more than 4,451 reported cases between 2010 and 2015 according to the Peruvian epidemiology directorate. However, little is known regarding the diversity and distribution of sand fly vectors in this region. In this study, we aimed to characterize the sand fly fauna in this endemic setting and identify sand fly species naturally infected with *Leishmania* possibly involved in pathogen transmission.

**Methods:**

Sand fly collections were carried out during 2014 and 2015 in the communities of Flor de Acre, Villa Primavera, Mavila and Arca Pacahuara using CDC light traps and Shannon traps. Collected specimens were identified and non-blood-fed females were selected for *Leishmania* infection screening using kinetoplastid DNA-PCR (kDNA-PCR) and nested Real time PCR for species identification.

**Results:**

A total of 10,897 phlebotomines belonging to the genus *Lutzomyia* (58 species) and *Brumptomyia* (2 species) were collected. Our study confirmed the widespread distribution and abundance of *Lutzomyia (Trichophoromyia) spp*. (24%), *Lu*. *whitmani* (19.4%) and *Lu*. *yucumensis* (15.8%) in the region. Analysis of Shannon diversity index indicates variability in sand fly composition across sites with Villa Primavera presenting the highest sand fly diversity and abundance. *Leishmania* screening by kDNA-PCR resulted in 45 positive pools collected from Flor de Acre (34 pools), Mavila (10 pools) and Arca Pacahuara (1 pool) and included 14 species: *Lu*. *yucumensis*, *Lu*. *aragoi*, *Lu*. *sallesi*, *Lu*. *sherlocki*, *Lu*. *shawi*, *Lu*. *walkeri*, *Lu nevesi*, *Lu*. *migonei*, *Lu*. *davisi*, *Lu*. *carrerai*, *Lu*. *hirsuta*, *Lu*. *(Trichophoromyia) spp*., *Lu*. *llanosmartinsi* and *Lu*. *whitmani*. *Lutzomyia sherlocki*, *Lu*. *walkeri* and *Lu*. *llanosmartinsi* had the highest infection rates (8%, 7% and 6%, respectively). We identified *Leishmania guyanensis* in two *Lu*. *whitmani* pools, and *L*. *braziliensis* in two *Lu*. *llanosmartinsi* pools and one *Lu*. *davisi* pool.

**Conclusions:**

Based on our collections there is high sand fly diversity in Madre de Dios, with differences in sand fly abundance and species composition across sites. We identified 14 sand fly species naturally infected with *Leishmania* spp., having detected natural infection with *L*. (*V*.) *guyanensis* and *L*. (*V*.) *braziliensis* in three sand fly species. These results suggest the presence of several potential vectors that vary in their spatial and geographical distribution, which could explain the high prevalence of CL cases in this region.

## Author summary

Leishmaniasis is a neglected disease that affects more than 2 million people worldwide. The identification of putative *Leishmania* vectors is an important step towards the design of better control strategies and estimating the risk of transmission in endemic areas. In this paper the authors explored the distribution of sand flies and identified potential vectors in a largely unexplored setting in the Southeastern Peruvian Amazon Basin. Three new sand fly species *Lutzomyia naiffi*, *Lu*. *dereuri* and *Lu*. *flabellata* are reported for Peru. In addition, they found fourteen sand fly species naturally infected with *Leishmania* that comprised seven new reports for Peru and one for the Americas. This information will serve as a baseline for future surveillance and intervention studies in this highly endemic area.

## Introduction

Leishmaniasis is a group of neglected tropical diseases caused by digenic protozoan of the genus *Leishmania*. This disease affects over 12 million people in more than 98 countries worldwide and causes more than 1.5 million cutaneous leishmaniasis (CL) new cases per year [1]. *Leishmania* parasites cause a wide spectrum of clinical manifestations that are divided into cutaneous, mucosal (ML) and visceral leishmaniasis (VL) [2, 3]. In the New World, CL is the most common clinical form of disease [1] leading to disfigurement, functional impairment and stigma in affected patients. CL is mainly caused by species of the *Viannia* subgenus, including *L*. *(Viannia) braziliensis*, *L*. *(V*.*) peruviana*, *L*. *(V*.*) guyanensis* and *L*. *(V*.*) panamensis* [4, 5] that are widely distributed in the Amazonian region.

The transmission cycle of leishmaniasis is highly dependent on the interaction of the sand fly vector and the mammalian host. In the New World, transmission occurs by the bite of infected phlebotomine sand flies of the genus *Lutzomyia*. Although, more than 500 sand fly species have been reported in the Americas, only 30 are known vectors of leishmaniasis [6, 7]. This evidence underscores the need to study the distribution and identification of possible *Leishmania* vectors.

Peru is among the ten countries that hold more than 75% of all CL infections worldwide [1] with 5,955 cases reported in 2015. The Amazonian region of Madre de Dios located near the border with Brazil is a highly endemic leishmaniasis area with an incidence rate of 9 cases per 10,000 person-years and contributing with up to 13% of all leishmaniasis cases in the country.

This high incidence in leishmaniasis cases in this and other Amazonian regions could be a result of the rich diversity of sand flies, leishmaniasis reservoirs and human driven activities like illegal mining, logging, and chestnut harvesting [8–10].

The presence of *Leishmania* infection in humans has been extensively documented in Madre de Dios with reports of *L*. *(V*.*) braziliensis*, *L*. *(V*.*) lainsoni* and *L*. *(V*.*) guyanensis* [5, 11–13]. However, information about *Leishmania* vectors, reservoirs, their role in disease transmission and the variables influencing their distribution is still limited [14–16]. For instance, a surveillance study using molecular methods for parasite identification, failed to detect *Leishmania* on more than 80 wild native rodents [16].

We conducted vector surveillance during 2014 and 2015 in different sites located in the region of Madre de Dios near the border with Brazil and employed molecular methods to identify natural *Leishmania* infections. Our results allowed us to characterize the dynamics of the sand fly populations and contributed to the understanding of pathogen transmission in the Southeastern Peruvian Amazon.

## Methods

### Ethics statement

This study (NAMRU6.2014.0007) was exempt from NAMRU-6 IRB review as this project did not involve humans as the subject of the study evaluation.

Therefore, this study did not meet the definition of research involving human subjects, and 32 CFR 219 does not apply. Sand fly collections were performed under approval from the General Directorate of Forestry and Wild Fauna from the Ministry of Agriculture and Irrigation of Peru (Resolución Directoral No. 0406-2013-MINAGRI-DGFFS/DGEFFS)

### Study sites for 2014

**Fig 1:**
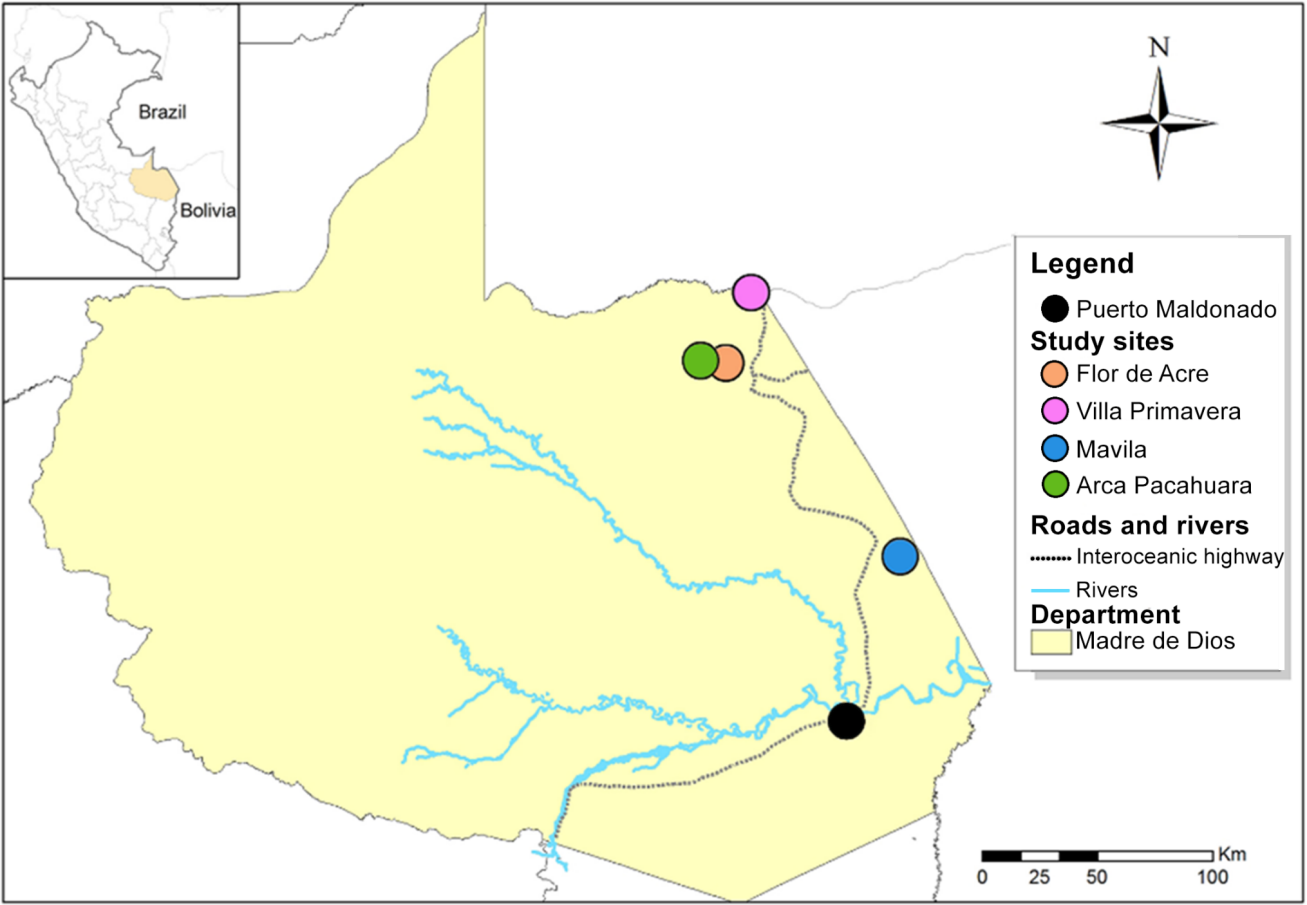
Map of the study area. This figure illustrates the Peruvian region of Madre de Dios and its capital city (Puerto Maldonado) crossed by the interoceanic highway. Collection sites are showed as points in the map as colored circles.

In 2014, sand flies were collected in February, May and September in the community of Villa Primavera (11° 02' 33.5"S, 69° 34' 24.6"W, 295 m.a.s.l.), and in May and September in the community of Flor de Acre (11° 19' 54.3"S, 69° 36' 20.6"W, 292 m.a.s.l) (**Fig 1**).

Flor de Acre was selected as a study site based on previous evidence of *Leishmania* vectors [17] whereas Villa Primavera was chosen due to its location at 13 km from the Brazilian border constituting a stopover.

### Study sites for 2015

In 2015, sand flies were collected in June and August in the communities of Flor de Acre, Arca Pacahuara (11° 19’ 57.6”S, 69° 36’ 53.8”W, 272 m.a.s.l.) and Mavila (11° 57’ 53.1”S, 69° 09’ 46.2”W, 206 m.a.s.l.) (**Fig 1**). The change in study sites was based on preliminary results from 2014 and reports of human CL in Arca Pacahuara and Mavila by the Peruvian Ministry of Health.

All study sites have a humid sub-tropical climate with annual temperatures between 19°C to 36°C with occasional low temperature periods that can reach 13°C. These sites have an annual precipitation of more than 3,000 mm that occurs mostly between January to April.

The sites are experiencing a rapid change of land use and deforestation due to illegal mining, agriculture, Brazil nut extraction, and livestock farming that are the major economic activities in the area.

### Sand fly collections and morphological identification

At each site, sand flies were collected inside, immediately outside and in the surrounding area of four households using six CDC light traps per site. Collections were conducted for 12 hours per night (18:00–06:00) during five consecutive nights. In addition, Shannon traps were used for collections outside and in the areas surround the houses from 18:00 to 21:00.

Sand flies were transported in 70% ethanol to the Entomology Department at NAMRU-6 in Lima where they were identified using keys developed by Young and Duncan [18] and Galati [19]. Female specimens were processed using a modified protocol to allow molecular analysis [17]. Briefly, the head and the two last abdominal segments were separated and placed in lactophenol for two hours at room temperature. These regions contain key taxonomic structures (cibarium, palpomeres, flagellomeres and spermathecae) which are used for species identification. The remaining parts of the sand fly were preserved in absolute ethanol at -20°C for molecular biology.

Male specimens were placed in 20% KOH for 12 to 24 hours at room temperature. Then, specimens were clarified with lactophenol for two hours at room temperature. Male and female specimens were mounted permanently on Euparal.

### DNA extraction and molecular detection of *Leishmania*

We selected non-blood-fed female sand flies that were pooled in sets of 1–10 specimens according to species, study site, household, collection date and trap type.

DNA from non-blood fed female sand flies was isolated using the DNeasy Blood & Tissue kit (QIAGEN) following the standard manufacturer’s protocol for isolation of insect genomic DNA.

In order to detect the presence of *Lutzomyia* DNA, we employed a PCR targeting the *Lutzomyia* 12S ribosomal DNA using primers T1B 5′-AAA CTA GGA TTA GAT ACC CT-3′ and T2A 5′-AAT GAG AGC GAC GGG CGA TGT-3′ as previously described [17, 20]. The reactions were prepared in 25 μL that contained1X Taq polymerase buffer (Invitrogen, Carlsbad, CA), 1.5 mM MgCl2, 125 μM dNTPs, 0.5 μM of each primer, 1 unit of Taq DNA polymerase (Invitrogen), and 5 μL of DNA sample. The PCR was run on a thermocycler under the following cycling conditions: initial denaturation at 94°C for 5 minutes followed by 35 cycles of denaturation 94°C for 20 sec, annealing at 56°C for 30 sec, and extension at 72°C for 25 sec; and a final extension step at 72°C for 5 min. This reaction generates a 360 bp product in the presence of sand fly DNA that serves to rule out the presence of PCR inhibitors in extracted DNA and as a positive control.

The presence of *Leishmania* DNA was detected by a PCR that targets the *Leishmania* minicircle [21–23]. This region is a high copy number DNA sequence present in the kinetoplast of *Leishmania* and other related protozoa that has been shown to be highly sensitive and specific [21, 23]. Reactions were carried out in 20 μL of PCR mixture containing 1X Taq polymerase buffer (Invitrogen, Carlsbad, CA), 1.5 mM MgCl_2_, 125 μM dNTPs, 0.5 μM of each primer, 1 unit of Taq DNA polymerase (Invitrogen), and 4 μL of DNA sample. The thermal cycling conditions consisted of an initial denaturation at 94°C for 5 min followed by 35 cycles of denaturation at 94°C for 45 sec, annealing at 58°C for 45 sec, and extension at 72°C for 60 sec; and a final extension step at 72°C for 5 min. This reaction generates a 120 bp amplification band that is considered positive for the *Leishmania* genus.

In order to detect the infecting *Leishmania* species on the kDNA positive samples we employed a FRET based nested Real Time PCR [24]. This method detects mutations on the 6PGD and MPI genes yielding different melting peaks according to the *Leishmania* species. For the first round of amplification, we prepared a 50μL reaction containing 1X Taq polymerase buffer (Invitrogen), 1.5 mM MgCl2, 200 μM dNTPs (Invitrogen), 0.8 μM or 1 μM of each primer (6PGD and MPI, respectively), 1.5 units of Taq DNA polymerase (Invitrogen), and 5 μL of DNA sample. The amplification setting consisted of an initial denaturation at 94°C for 5min followed by 35 cycles of denaturation at 94°C for 45 sec, annealing at 57°C (for MPI) or 62°C (for 6PGD) for 45 sec, and extension at 72°C for 90 sec; and a final extension at 72°C for 7 min for MPI or 5 min for 6PGD.

The second amplification round was performed on a 20μL reaction for each gene containing 1X LightCycler 480 Genotyping Master (Roche, Indianapolis, IN), 1.25 μM of forward primer, 0.25 μM of reverse primer, 0.75 μM of anchor and sensor probes, and 5 μL of PCR product from the first reaction.

The amplification setting was performed on a LightCycler 480 and consisted of an initial denaturation at 95°C for 5 min followed by 45 cycles of denaturation at 95°C for 10 sec, annealing at 60°C for 20 sec under a single acquisition step) and extension at 72°C for 20 sec. A melting curve analysis was performed at the end of the amplification cycles by heating the amplicons at 95°C for 10 sec, cooling at 50°C for 59 sec and then gradually increasing the temperature to 80°C with one acquisition step each °C.

Melting curves were analyzed using the LightCycler 480 Software Version 1.0 as previously described [24].

### Data analysis

We calculated the Shannon-Wiener (H) diversity index in PAST v3.12[25] for each study site using the equations $H=-\sum_{i=1}^{n}p\;\ln\;p$, where “p” represents the proportion in which each species “n” was collected (∑p = 1). The Hutcheson *t*-test was employed to assess the statistical significance of differences in Shannon diversity indexes between study sites [26].

Species abundance was calculated in Excel 2010 (Microsoft) using the Index of Species Abundance (ISA) [27] using the formula *ISA* = (a + Rj)/*k*. Briefly, for each site we established a rank of abundance from 1 (the species with the highest value) to the number of species collected (leaving in blank species not represented in the site or using the average for ties between 2 or more species). Then, we calculated “a” as the number of zero observations for each species in all sites multiplied “c” which is the single largest rank in all the data set plus 1. The value of Rj corresponds to the sum of ranks for a given species in all the sites whereas “k” corresponds to the number of sites. The resulting ISA values were converted into the Standardized Index of Species Abundance (SISA) using the formula *SISA* = (*c* − *ISA*)/(*c* − 1).

## Results

During the two years of the study, we collected 10,897 sand flies belonging to the genus *Lutzomyia* (10,800 specimens, 99.1%) and *Brumptomyia* (97 specimens, 0.9%). The majority of specimens were collected in the areas surrounding houses (76.57%), followed by primary forest patches (14.44%) and inside of the houses (8.99%).

Overall, we identified 58 *Lutzomyia* species and two *Brumptomyia* species with three new sand fly reports for Peru; *Lu*. *naiffi*, *Lu*. *dereuri* and *Lu*. *flabellata* that were collected in Flor de Acre (**S1 and S2 Tables**).

We were not able to identify the female specimens of the subgenus *Trichophoromyia* up to the species level due to the high similarity among females of this subgenus and the fact that we detected five distinct *Lutzomyia* (*Trichophoromyia*) species based on male morphology (*Lu*. *auraensis*, *Lu*. *loretonensis*, *Lu*. *clitella*, *Lu*. *nemorosa* and *Lu*. *ubiquitalis*). The same situation occurs for the subgenus *Pressatia* with three distinct species (*Lu*. *choti*, *Lu*. *calcarata* and *Lu*. *triacantha*).

*Lutzomyia* (*Trichophoromyia*) *auraensis* (male specimens), *Lutzomyia* (*Trichophoromyia*) *spp* and *Lu*. *davisi* were the most abundant species in the study sites (SISA = 0.98, 0.95, 0.93 for 2014 and 0.99, 0.97 and 0.95 for 2015, respectively). These species are suspected vectors of leishmaniasis and may have an important role in its transmission in the area.

### Sand fly collections of 2014

In February 2014, we collected 311 specimens in Villa Primavera with *Lu*. *(Trichophoromyia) spp*. (n = 239, 76.8%), *Lu*. *davisi* (n = 20, 6.43%) and *Lu*. *shawi* (n = 10, 3.22%) as the most abundant species out of 24 recorded (**S1 Table**). Collections were not performed in Flor de Acre during this month due to extreme weather conditions in the area.

In May 2014, we collected 4,629 specimens, 4.99% in Villa Primavera and 95.01% in Flor de Acre (**S2 Table**). The most abundant species on this collection were *Lu*. *(Trichophoromyia) spp* (n = 156, 67.5%) and *Lu*. *davisi* (n = 33, 14.2%) out of 24 species recorded in Villa Primavera; and *Lu*. *yucumensis* (n = 1,564; 35.6%) and *Lu*. *(Trichophoromyia) spp* (n = 2,158; 49.1%) out of 18 species recorded in Flor de Acre.

In September 2014, the number of collected specimens was lower than May with 2,643 specimens, 2.72% in Villa Primavera and 97.28% in Flor de Acre. The most abundant species were *Lu*. *aragoi* (n = 17, 23.6%) in Villa Primavera and *Lu*. *whitmani* (n = 2,033; 79.07%) in Flor de Acre, out of 16 and 34 species recorded, respectively.

Flor de Acre had a higher phlebotomine species richness (44 species in 5,999 specimens) than Villa Primavera (36 species in 410 specimens). However, Villa Primavera presented a significantly (p<0.05) higher Shannon diversity index (H = 2.15±0.16) than Flor de Acre (H = 1.72±0.31) due differences in the number of collected specimens.

### Sand fly collections of 2015

In June 2015, we collected 2,948 specimens; 18.69% in Arca Pacahuara, 16.82% in Flor de Acre and 64.48% in Mavila. Out of 9 species recorded, *Lu*. *(Trichophoromyia) spp* stood out as the most predominant species on the three sites (**S2 Table)**.

In August 2015 we collected 366 specimens, 18.58% in Arca Pacahuara (9 species), 18.03% in Flor de Acre (16 species) and 63.39% in Mavila (21 species). This low number of specimens could be the result of a drop in rainfall from June towards September.

Out of all species recorded, the most predominant species were *Lu*. *(Trichophoromyia) spp* in Arca Pacahuara (n = 53, 77.9%), *Lu*. *whitmani* in Flor de Acre (n = 16, 24.24%) and *Lu*. *davisi* (n = 56, 24.14%) in Mavila (**S2 Table**).

Mavila presented a higher species richness (33 species in 1,240 specimens) than Flor de Acre (22 species in 461 specimens) and Arca Pacahuara (13 species in 346 specimens). However, Flor de Acre had a significantly (p<0.05) higher Shannon diversity index (H = 1.79±0.12) than Arca Pacahuara (H = 0.69±0.14) and Mavila (H = 1.62±0.08).

### Sand flies naturally infected with *Leishmania*

Female sand fly specimens were grouped into 850 pools based on species; trap type, month of collection and site. The 12S ribosomal DNA PCR confirmed the presence of sand fly DNA on all samples and served as an internal control for DNA quality by ruling out the presence of PCR inhibitors.

**Fig 2:**
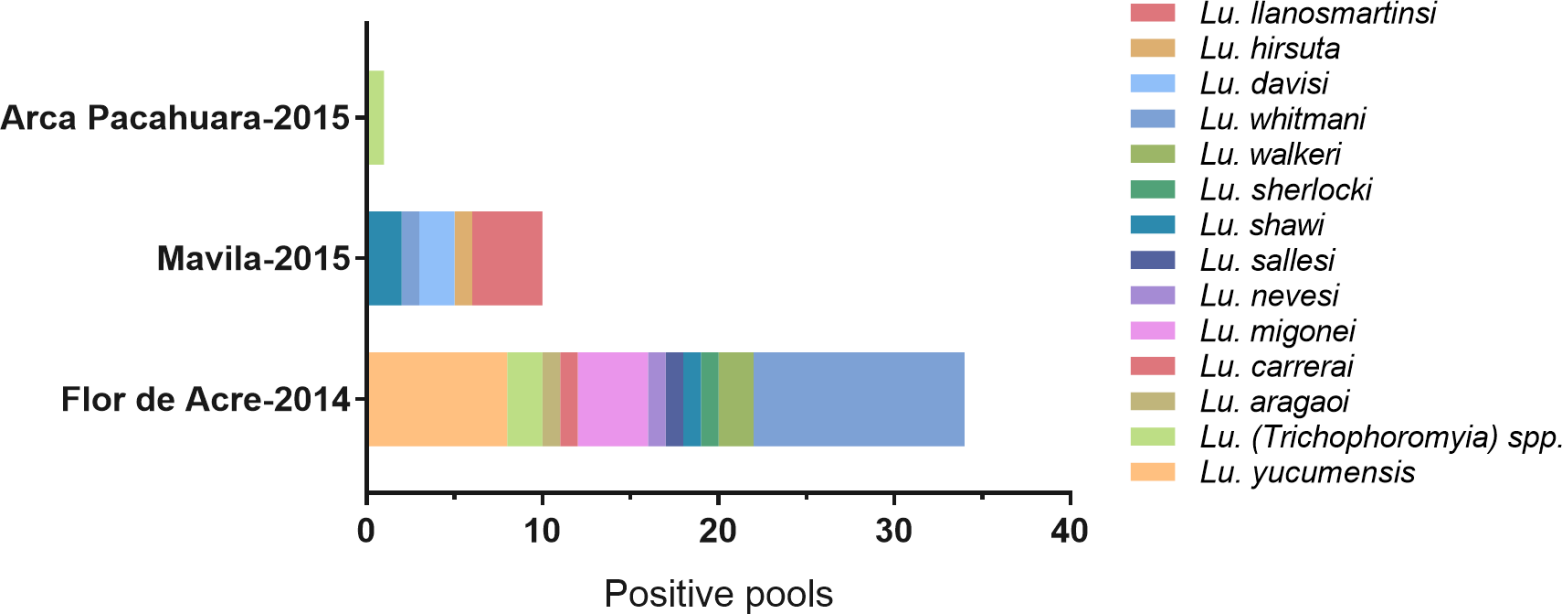
Positive pools detected by kDNA PCR. The figure shows that Mavila and Flor de Acre presented the highest number of positive pools. These pools belonged to up to 14 different *Lutzomyia* species.

*Leishmania* specific kinetoplast PCR detected parasite DNA on 45 pools from 14 different *Lutzomyia* species (**S3 Table, Fig 2**). Pools collected in Flor de Acre in 2014 and Mavila in 2015 accounted for 75.6% (n = 34) and 22.2% (n = 10) of all positives.

The group of sand fly species where we detected natural *Leishmania* infections in our study accounted for nearly 70% of all phlebotomines collected, and within this group *Lu*. *(Trichophoromyia) spp*, *Lu*. *whitmani*, *Lu*. *yucumensis* and *Lu*. *davisi* stood out as the predominant species (**Fig 2**).

The estimated minimum infection rates in 2014 for *Lu*. *whitmani*, *Lu*. *yucumensis*, *Lu*. *(Trichophoromyia) spp*. and *Lu*. *carrerai carrerai* were 1.09, 0.97%, 0.22% and 0.81%, respectively. In 2015, the minimum infection rates *Lu*. *(Trichophoromyia) spp* and *Lu*. *davisi* were 0.21% and 0.29%, respectively (**S4 Table**).

FRET-based Real-Time PCR was employed to identify the species of *Leishmania* present in the kDNA-positive pools. This assay confirmed the presence of *Leishmania (Viannia) guyanensis* in two pools of *Lu*. *whitmani* collected from Flor de Acre (**S3 Table**). Additionally, we detected *L*. *braziliensis* in two pools of *Lu*. *llanosmartinsi* and on one pool of *Lu*. *davisi* collected from Mavila in 2015 (**S3 Table**). We could not identify the *Leishmania* species in the remaining kDNA-positives due to lack of detectable amplification product on the Real-Time PCR assay.

To assess the relation of potential vector versus non-vector species we estimated their ratio for each locality. Our results indicate that Flor de Acre presented the highest potential vector versus non-vector ratio (2.3:1) **(Table 1).**

**Table 1:** Ratio of vector versus non-vector species per site (V:N). The table shows the number of different vector species collected at each study location, the ratio versus non-vector species and the predominant vector found at each site.

Study site	Year	Ratio (V:N)	Vector species	Collected vectors (%)	Predominant vector species (♀>100)
**Flor de Acre**	2014	2.3:1	14	69.9%	*Lu*. *whitmani*, *Lu*. *yucumensis*, *Lu*. *auraensis*, *Lu*. *(Trichophoromyia) spp*, *Lu*. *davisi*, *Lu*. *carrerai*, *Lu*. *migonei*
	2015	1.9:1	12	66.2%	*Lu*. *auraensis*, *Lu*. *davisi*
**Arca Pacahuara**	2015	0.62:1	8	38.3%	*Lu*. *auraensis*, *Lu*. *(Trichophoromyia) spp*.
**Mavila**	2015	0.7:1	10	40.5%	*Lu*. *auraensis*, *Lu*. *(Trichophoromyia) spp*, *Lu*. *davisi*
**Villa Primavera**	2014	0.7:1	10	39.8%	*Lu*. *(Trichophoromyia) spp*, *Lu*. *davisi*

**Fig 3:**
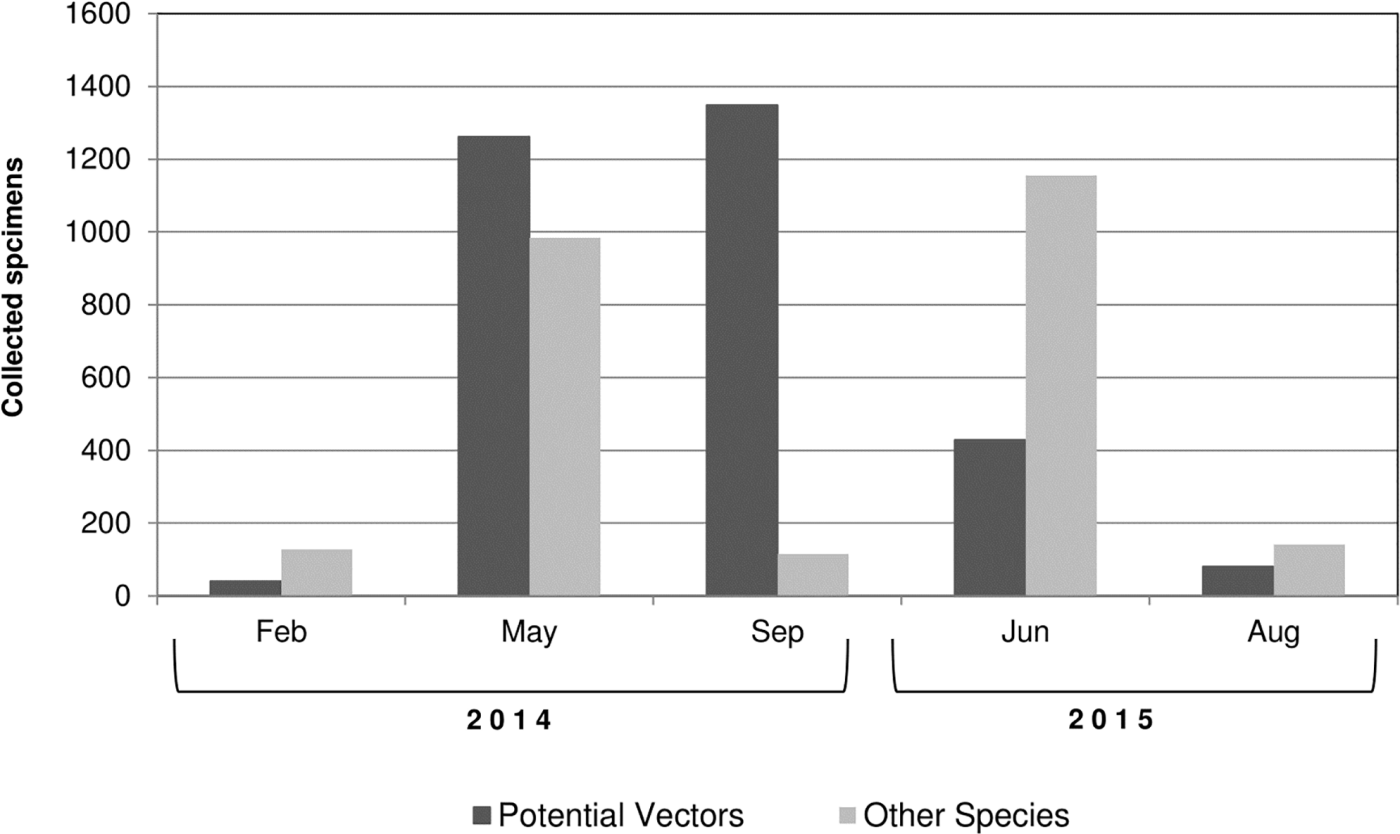
Relative abundance of sand fly specimens. This figure shows changes in sand fly abundance of putative vectors and non-vectors by month of collection.

The majority of phlebotomines were collected in May, September of 2014 and June 2015 (42.48%, 24.25% and 27.05%, respectively). In the other hand, a lower sand fly density was found during February and August (2.85% and 3.36% of the total, respectively). Putative *Leishmania* vectors were overrepresented in May, September and June (**Fig 3**).

## Discussion

The Peruvian region of Madre de Dios is an important focus of CL in Peru with multiple *Leishmania* species reported in humans [5]. This area has been experiencing a dramatic change in land use due to the increase of agriculture, logging and economic activities that are related to the trans-oceanic highway that connects Peru, Brazil and Bolivia. This economic boost has led to the appearance of new communities along this highway such as Arca Pacahura and Mavila that are mainly formed by immigrants from nearby Peruvian regions (Cusco, Puno and Arequipa) [28].

It is known that leishmaniasis is highest among people living near to forest edges or working in forested areas [29]. In this sense, the colonization of previously forested areas has resulted in the rapid emergence and spread of CL cases in Madre de Dios placing this disease as an important public health problem.

The study sites have environmental variables that favored a similar sand fly biodiversity to those found in low latitude areas of Central America and South America where 1 ha of forest can contain up to 50 different species [30]. The number and recorded species collected in our study support this high biodiversity which is similar to previous reports from the region of Acre on the Brazilian side of the Peru-Brazil border [9, 10, 31].

Our results show that there is important variation in the number of species across sites and time of collection (**S1 Table**). However, members of the *Trichophoromyia* and *Psychodopygus* subgenus are consistently abundant in all study sites according to the SISA estimates (**S1 and S2 Tables**).

Differences in abundance and diversity between study sites could result in different risks of *Leishmania* transmission. In this regard, our results of 2014 showed the extent of this variation with up to 7,000 sand flies collected in Flor de Acre versus only 600 in Villa Primavera. On the same year, *Lu*. *whitmani* was the predominant species on Flor de Acre accounting with 30% of all collected individuals whereas in Villa Primavera it represented only 0.8% of all collected sand flies.

This difference in collected specimens could be a result of the different degrees of deforestation in the two sites. Villa Primavera is a small village on a highly deforested area near the transoceanic highway while Flor de Acre is located far from the highway and surrounded by a primary forest.

Interestingly, the sites studied in 2015 presented a different sand fly composition from the ones in 2014. In these areas, *Lu*. *(Trichophoromyia) spp*. accounted for the majority of specimens collected (90% in Arca Pacahuara, 73% in Mavila and 46% in Flor de Acre). Among these sites, Mavila accounted for the majority of collected sand flies (2,133 versus 619 in Arca Pacahuara and 562 in Flor de Acre). The presence of primary forested areas appeared to be related to these differences given the location of Mavila in the deep jungle.

Our study has also shown that abundance and sand fly diversity can vary in the same area, potentially complicating control activities due to differences in sand fly behavior. In this regard, *Lu*. *yucumensis* and *Lu*. *(Trichophoromyia) spp* were the most abundant species in Flor de Acre in May 2014. However, their abundance decreased towards September 2014 and *Lu*. *whitmani* replaced them as the dominant species.

Regardless of these variations, the most abundant species at each site are suspected putative *Leishmania* vectors with confirmed PCR infection (*Lu*. *(Trichophoromyia) spp*., *Lu*. *whitmani*, *Lu*. *yucumensis*, and *Lu*. *davisi*). However, less prevalent species presented higher minimum infection rates such as *Lu*. *sallesi/cortelezzi* (14.29%), *Lu*. *walkeri* (12.5%) and *Lu*. *sherlocki* (9%) (**S4 Table**).

The abundance of the subgenera *Trichophoromyia*, *Psychodopygus* and *Nyssomyia* is consistent with previous studies conducted in Peru and Brazil [10, 17, 31] and indicates that species from these subgenera are predominant in the Peruvian and Brazilian Amazon Basins and could play and important role in leishmaniasis transmission in the region.

In terms of natural *Leishmania* infection, our study has shown that the proportion of infected sand flies without considering *Lutzomyia* species is not statistically different across sites according to the Fisher exact test. This suggests that variations in *Leishmania* transmission at each site will likely depend on the behavior and vector competence of the predominant species rather than differences in the prevalence of *Leishmania*.

Infection with *Leishmania (V*.*) guyanensis* in *Lu*. *whitmani* and *L*. *(V*.*) braziliensis* in *Lu*. *llanosmartinsi* and *Lu*. *davisi* suggests a role for these species in leishmaniasis transmission in Peru and Brazil [31]. This finding is further supported by the isolation of these two *Leishmania* species from tissue biopsies from patients with CL in Madre de Dios [5, 12, 13].

Our minimum infection rates for *Lu*. *davisi* and *Lu*. *(Trichophoromyia) spp*. are similar to the ones obtained in a previous study conducted in 2010 on Flor de Acre [17] suggesting that the composition of potential vectors has remained constant in this area. However, our infection rates differ from other studies conducted in the neighboring Brazilian state of Acre. In this state, *Lu*. *davisi* and *Lu*. *(Trichophoromyia) spp*. presented higher infection rates whereas *Lu*. *whitmani* presented a lower infection rate than in our study (1.84, 2.05 and 0.5%, respectively) [10, 31].

It is important to note that *Lu*. *whitmani* has been suggested as one of the most important vectors of CL in various regions of Brazil [32]. This species is highly anthropophilic and has been frequently found in areas undergoing deforestation. Previous epidemiological assessments from deforested areas indicate that transmission of leishmaniasis relies mainly on this species [32–34].

This is the first report of natural *Leishmania* infection in *Lu*. *sherlocki* and the first report of seven sand fly species naturally infected with *Leishmania* in Peru: *Lu*. *whitmani*, *Lu*. *sherlocki*, *Lu*. *llanosmartinsi*, *Lu*. *shawi*, *Lu*. *yucumensis*, *Lu*. *nevesi* and *Lu*. *walkeri*. These species are widely distributed in the Amazon Basin underscoring the need to assess their vectorial competency [9, 17, 31]. In this regard, future studies will be oriented towards the identification of the infecting *Leishmania* species in the kDNA positive/ Real Time PCR negative samples employing alternative methods.

Although we did not identify *Lu*. *longipalpis* during the study execution, we collected specimens of *Lu*. *migonei* in Flor de Acre and Mavila. This species has been reported as a confirmed vector of *L*. *(L*.*) infantum* [35] highlighting a potential risk for the introduction of visceral leishmaniasis into this region.

An important limitation to consider for this study is that the finding of *Leishmania* DNA in a sand fly species is not a conclusive evidence of its role as vector. Vectorial role is confirmed by a series of criteria that include the capacity of the sand fly to maintain and transmit the parasite and the isolation of the same parasite strain from the human and the mammalian reservoir in the focus [6, 36]. Further studies should be oriented towards assessing the vectorial competency of putative vectors identified on this study.

Leishmaniasis control is highly dependent on the understanding of the ecology and disease epidemiology in endemic settings. In this sense, sand fly surveillance and identification of natural *Leishmania* infection are critical to assess the role of each species and design better and efficient control strategies [6, 36].

Our results underscore the need for increased control efforts against the sand fly vectors in the area and shed light into the potential effects of human activities in the epidemiology of the leishmaniasis in the Peruvian Amazon Basin.

## Supporting information

S1 Table Sand fly species collected in Madre de Dios during 2014. (XLSX)

S2 Table Sand fly species collected in Madre de Dios during 2015. (XLSX)

S3 Table Positive sand fly tools for Leishmania by kDNA and Real Time PCR. (XLSX)

S4 Table Minimum infection rates of positive sand flies. (XLSX)
